# Supplementary material for: Elimination of Vitamin D Signaling Causes Increased Mortality in a Model of Overactivation of the Insulin Receptor: Role of Lipid Metabolism
Source: Nutrients. 2022 Apr 5;14(7):1516. doi: 10.3390/nu14071516 (PMC9002971; doi:10.3390/nu14071516)

Supplementary tables

Supplementary Table S1. PCR oligonucleotides.

Gene	Sequence
**PTEN**	FWD: CAAGCACTCTGCGAACTGAG
	REV: AAGTTTTTGAAGGCAAGATGC
**Cre-ER^{TM}**	FWD: ACGAACCTGGTCGAAATCAGTGCG
	REV: CGGTCGATGCAACGAGTGATGAG
**VDR wt**	FWD: TTTGGCCTTTCTGCTTGCCTCTTC
	REV: TCAGTTGATATCCCTGGGAGGC
**VDR LoxP**	FWD: TTTGGCCTTTCTGCTTGCCTCTTC
	REV: AGCGACACTCTTGGTCTGGTTCC
**∆PTEN excised**	FWD: ACTCAAGGCAGGGATGAGC
	REV1: AATCTAGGGCCTCTTGTGCC
	REV2: GCTTGATATCGAATTCCTGCAGC
**∆VDR excised**	FWD: CACAACAGTCAGAGGCAGTAAGCAAAGC
	REV1: AGCGACACTCTTGGTCTGGTTCC

Supplementary Table S2. Real time qPCR primers.

Gene	Sequences	bp	ID
**TBP**	FWD: AAA ATG GTG TGC ACA GGA GCC	141	NM_013684.3
	REV: CAC ATC ACA GCT CCC CAC CAT		
**CEBPA**	FWD: GTGGAGACGCAACAGAAGGT	136	NM_001287514.1
	REV: CCTTGACCAAGGAGCTCTCA		NM_007678.3
**PEPCK**	FWD: CCAGTGCCCCATTATTGAC	249	NM_011044.3
	REV: CCGAAGTTGTAGCCGAAGAA		
**G6PC**	FWD: ACTTTCCCCACCAGGTCGT	169	NM_008061.4
	REV: ACCCCTAGCCCTTTTAGTAGCA		
**PGC1A**	FWD: GATCACGTTCAAGATCGCCCTAC	178	NM_008904.2
	REV: TAAATCACACGGCGCTCTTC		
**PPARA1**	FWD: ATTTGGGCGTATCTCACCG	248	NM_011144.6
	REV: GGACTTTCCAGGTCATCTGC		
**CPT1**	FWD: ACATCGTGAGTGGCGTCCT	171	NM_013495.2
	REV: GACCCGAGAAGACCTTGACCATA		
**ACOX1**	FWD: TAACTTCCTCACTCGAAGCCA	283	NM_015729.3
	REV: AGTTCCATGACCCATCTCTGTC		
**GLUT2**	FWD: TTGTCATCGCCCTCTGCT	228	NM_031197.2
	REV: CACTCTCTGAAGACGCCAGGAA		
**FGF21**	FWD: TGAAGCCAGGGGTCATTCAA	203	NM_020013.4
	REV: GTTTGGGGAGTCCTTCTGAGG		

Supplementary Table S3. Primary and secondary antibodies.

Protein	Company	Cat. Nº	Host	Reactivity	Dilution
**PTEN**	Cell Signaling	9188	Rabbit	H,M,R	1/1000
**VDR**	Santa Cruz	sc-1008	Rabbit	H,M,R	1/1000
**pAkt**	Cell Signaling	4060S	Rabbit	H,M,R	1/1000
**Akt**	Santa Cruz	sc-5298	Mouse	H,M,R	1/1000
**GAPDH**	BioLegent	919501	Mouse	H,M,R	1/1000
**anti-mouse IgG-HRP**	Jackson Immuno Research	115-035-003	Goat	Secondary antibody	1/10.000
**anti-rabbit IgG-HRP**	Cell Signaling	7074	Goat	Secondary antibody	1/10.000

Supplementary Figure S1. Mouse genotypes and target protein expressions. The presence of the excised exons (A) or the protein expressions (B) were determined. The relative amount of protein was also quantified (C). * p<0.05 vs CNT; ** p<0.01 vs CNT. CNT: Control. VDR-KO: Vitamin D receptor knockout. PTEN-KO: Phosphatase and tensin homolog knockout. DKO: Double knockout.

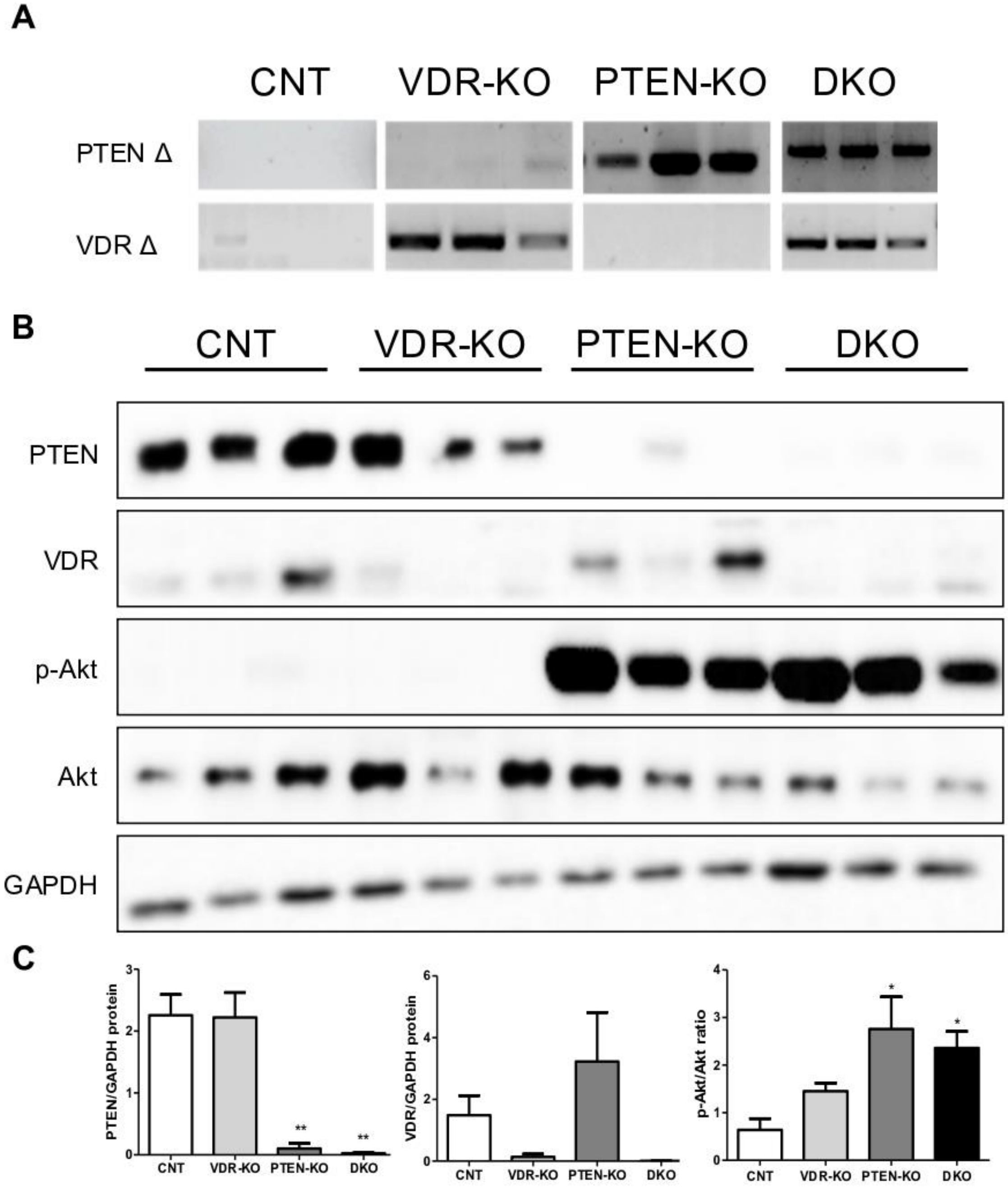

Supplement: Supplementary file 1 [file nutrients-14-01516-s001.zip › nutrients-1660092-supplementary.pdf]
